# Clinical outcomes in metastatic prostate adenocarcinoma treated with abiraterone and enzalutamide

**DOI:** 10.3332/ecancer.2024.1763

**Published:** 2024-09-11

**Authors:** Misbah Younus Soomro, Saqib Raza Khan, Hashim Ishfaq, Insia Ali, Mirza Rameez Samar, Arif Hameed, Nawazish Zehra, Munira Moosajee, Yasmin Abdul Rashid

**Affiliations:** 1 Department of Oncology, Section of Medical Oncology, Aga Khan University Hospital, Sindh, Karachi 748000, Pakistan; 2 Aga Khan Medical College, Aga Khan University Hospital, Sindh, Karachi 748000, Pakistan

**Keywords:** prostate cancer, metastasis, enzalutamide, abiraterone, outcomes

## Abstract

**Aim:**

The management of metastatic prostate cancer has progressed immensely in the last decade. This study aims to investigate the real-world clinical outcomes of metastatic prostate adenocarcinoma treated with abiraterone and enzalutamide. The findings will assist healthcare providers in making more informed decisions when choosing between these two drugs for treating these patients.

**Methods:**

A retrospective analysis of 80 patients at our tertiary care hospital was conducted from January 2015 to July 2022. Data were analysed using SPSS version 20.0. An independent sample *T*-test was used for continuous data and the chi-square test for categorical data. Medians and means were calculated for continuous or ordinal variables. Kaplan-Meier survival curves presented progression-free and overall survival (OS), with comparisons made using the log-rank test. Survival rates with 95% CIs were reported, with *p* < 0.05 considered significant.

**Results:**

In our final analysis of 80 patients, the median age was 65 years, with 88% having an eastern cooperative oncology group performance status between 0 and 2. Histopathology showed adenocarcinoma in 91% of cases. Grade Group III–IV disease was present in 51.3%, and 67.5% had a Gleason Score of >8. Bilateral orchidectomy was performed in 41 patients (51.25%), with a median Gonadotropin-releasing hormone analogue use of 32 months. Most patients (72.5%) were castration-sensitive. Among the 80 patients, 60 (75%) were treated with abiraterone and 20 (25%) with enzalutamide. The prostate-specific antigen (PSA) doubling time was >6 months in 80% of the abiraterone group and 75% of the enzalutamide group. PSA response rates were similar for both drugs, with comparable rates of progressive disease, partial response, stable disease and complete response (*p* = 0.036). There was no significant difference in median time to progression (19 months for abiraterone versus 18 months for enzalutamide) (95% CI 9.7–27.9; *p* = 0.004). The median OS for the entire cohort was 67 months (95% CI 39–94; *p* = 0.003).

**Conclusion:**

The findings suggest that both abiraterone and enzalutamide are effective in prolonging progression-free and overall survival in this patient population, providing comparable safety. Further studies are recommended to validate these findings and inform clinical decision-making.

## Highlights

1. Abiraterone and enzalutamide demonstrated similar effectiveness in treating metastatic prostate adenocarcinoma.
2. No differences between abiraterone and enzalutamide treatments were observed regarding time to treatment progression, prostatespecific antigen response or progression type.
3. Both drugs provided clinically significant survival benefits.

## Introduction

The management of prostate cancer has progressed immensely in the last decade. More than 4,000 new cases of prostate cancer are diagnosed every year in Pakistan with 1.9% of all cancer-related deaths [1]. In metastatic disease, treatment modalities include hormonal treatment, cytotoxic chemotherapy and targeted therapy. Abiraterone and enzalutamide are frequently used as first-line antiandrogen therapy in metastatic castration-sensitive prostate cancer (mCSPC) as well as metastatic castrate-resistant prostate cancer.

Abiraterone is a CYP17A1 blocker and blocks testicular, tumoural and adrenal androgen production in both castrate and non-castrate men. Multiple clinical trials have shown survival benefits of abiraterone in metastatic disease in patients with treatment naïve as well as among those who are previously treated with other systemic treatments [2]. Enzalutamide, on the other hand, is an androgen receptor antagonist. It is currently approved in castration-sensitive and castration-resistant metastatic prostate cancer. Furthermore, it has also proven to increase overall survival (OS) in randomised control trials and hence is now recommended in upfront settings [3, 4].

The current standard of care in metastatic prostate cancer includes androgen deprivation therapy (ADT) along with either intravenous systemic chemotherapy (docetaxel) or oral hormonal agents. The preferred oral hormonal agents include frequently used abiraterone, enzalutamide and newer agents; apalutamide and darolutamide. Among the frequently used hormonal agents, there is no consensus in preferring one over the other in the first-line setting. A meta-analysis was done to review the clinical outcome of these agents. They reported a preference for abiraterone followed by enzalutamide but since there are no prospective clinical phase III trials with head-to-head comparisons, both of these agents are used in first-line settings, as per the physicians’ decision [5].

We propose a single-centre, retrospective analysis of metastatic prostate adenocarcinoma from January 2015 to July 2022, to see the outcomes of patients treated with enzalutamide and abiraterone in our region. As the prevalence of these patients in Pakistan is on the rise, understanding the effectiveness of these two drugs in this population is crucial. Currently, there is no data on the activity of these drugs and side effect profiles in our population; hence, this study can help healthcare providers make more informed decisions when choosing between these two drugs for the treatment of such patients.

## Materials and methods

To investigate the effect of first-line hormonal treatment on outcomes of metastatic prostate cancer cases, we conducted a retrospective cohort study at the Department of Medical Oncology in our tertiary care hospital, in Karachi, Pakistan. This study was approved by the hospital Ethical Review Committee (ERC) and subsequently, medical records of known prostate cancer patients were reviewed from January 2015 to July 2022.

**Figure 1.**
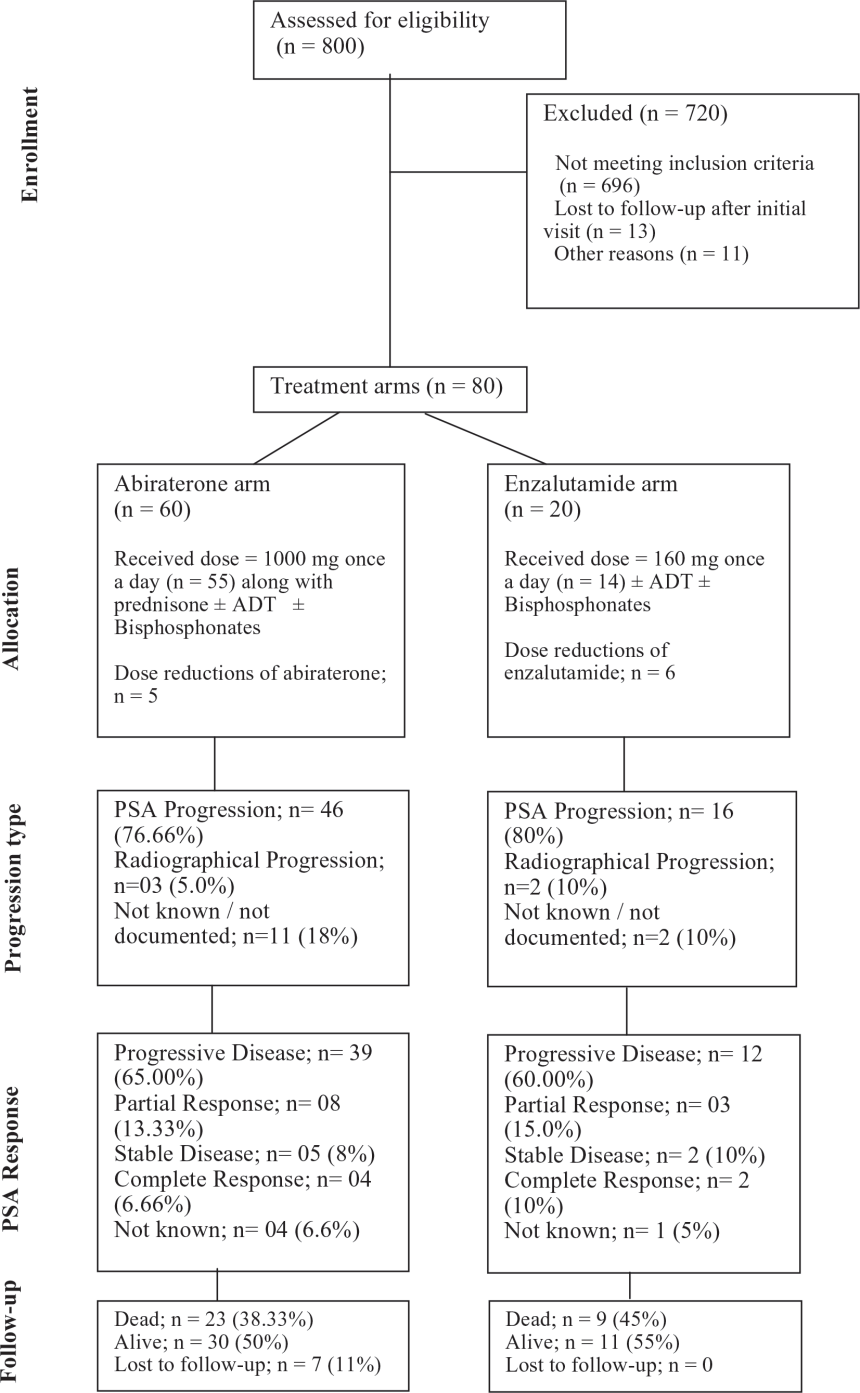
Consort diagram showing the flow of participants through each stage of the study.

We employed a non-probability purposive sampling methodology for patient selection. We chose a non-probability purposive sampling methodology to ensure that our study focused on a specific subset of prostate cancer patients who met our inclusion criteria. Although all prostate cancer patients were considered, only those treated with first-line abiraterone or enzalutamide were included in the final analysis. This approach allowed us to comprehensively analyze the outcomes of these specific treatments in a real-world setting. Patients with a) other coexistent or historical malignancies, b) predominant neuroendocrine prostate cancer pathology and c) patients who were treated with either ADT alone or other hormonal agents or received systemic chemotherapy upfront; were excluded from the study analysis. Patients with metastatic prostate cancer who were treated with either first-line abiraterone or enzalutamide with or without ADT were included. Of the 800 patient files reviewed, 80 fulfilled our study inclusion criteria and were selected for further analysis (Figure 1).

Medical records were reviewed to obtain patients’ baseline characteristics at diagnosis. These include age, eastern cooperative oncology group (ECOG) performance status, comorbidities, family history of malignancy, radiographical imaging study which includes computed tomography chest, abdomen and pelvis, bone scan, positron emission tomography (PET) scan, prostate-specific membrane antigen (PSMA) PET scan as well as haematological parameters, and biochemical values including serum concentration of prostate-specific antigen (PSA), testosterone and lactate dehydrogenase (LDH). We evaluated therapeutic interventions relevant to the patient’s prostate cancer management such as if they had a) undergone surgical procedures such as radical prostatectomy or orchidectomy; b) been administered Gonadotropin-releasing hormone (GnRH) analogues and duration of use; c) used bone-protection agents; i-e bisphosphonates and d) been subjected to radiation therapy.

The prostate biopsy histopathology reports established tumour characteristics such as the Gleason grade, group, score, pattern and risk stratification. Metastatic status and sites of spread were determined. We also determined patients’ vital status, whether they were alive, deceased or lost to follow-up.

Patients were later designated into two categories: those treated with abiraterone (1,000 mg orally once daily along with prednisone 5 mg orally) and those treated with enzalutamide (160 mg orally once daily) with or without ADT and bisphosphonates. The patients were being followed in the clinic at regular intervals with repeat serum PSA levels and subsequent scans for response assessment. The primary endpoint established was time to progression which was defined as the time from initiation of treatment to either clinical or radiographical progression or death due to prostate cancer, whichever occurs first.

The patterns of progression which include PSA progression and radiographical progression were also documented. The PSA progression is defined as an increase of 25% or more and an absolute rise of at least 2 ng/mL above the nadir, confirmed by a subsequent value measured 3–4 weeks later. The radiographic progression was assessed by an independent central radiology review, utilising the response evaluation criteria in solid tumours for soft tissue and criteria adapted from the prostate cancer clinical trials working group 2 for bone disease.

The PSA response, a secondary endpoint, was classified into partial response (PR), complete response (CR), progressive disease (PD) and stable disease (SD). A PR was defined to be a PSA decline of greater than 50%, while a CR indicated undetectable PSA levels. SD was characterised by consistent PSA without a clinically significant increase or decrease in PSA levels, and PD indicated a PSA rise exceeding 25%. PSA doubling time was also evaluated and was defined as the number of months it would take for PSA to increase two-fold, and was determined during the treatment period for each patient. We also sought to determine progression-free survival (PFS) in both treatment arms. The OS in the entire patient cohort was also reported.

### Statistical analysis

Data were analysed in SPSS version 20.0. For continuous data, we used an independent sample *T*-test and categorical data, was analysed through the chi-square test. The median was calculated for continuous or ordinal variables, while the mean was calculated for continuous variables. Categorical variables were summarised using counts and percentages. Kaplan-Meier survival curves were performed to present patients’ PFS and OS. Log-rank test was used to compare median survival times. Survival rates with their corresponding 95% CIs were reported. A *p*-value of <0.05 was considered statistically significant.

## Results

In our final analysis, 80 patients were evaluated. The median age was 65 years and most patients had an ECOG performance status between 0 and 2, accounting for 88% of the cohort, while a smaller subset displayed a status of 2–4. The group was characterised by a high prevalence of comorbid conditions, with diabetes mellitus present in 36%, hypertension (HTN) in 57% and ischemic heart disease (IHD) in 30%. The patients reported a diverse familial cancer history, with incidences of patients with a positive family history at 12.5%. Genetic testing was conducted on 13.25% of the patient population. Among these, two patients tested positive for the BRCA gene mutation and four patients were found to have mutations of unknown significance (Table 1).

**Figure 2.**
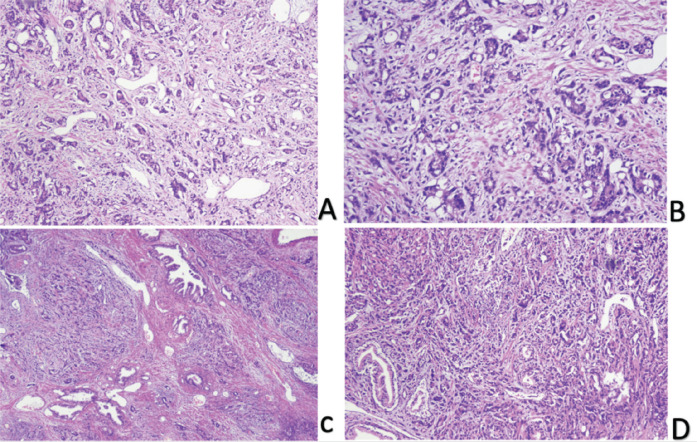
Prostate acinar adenocarcinoma.

Tumour characteristics were also evaluated. Histopathology predominantly showed adenocarcinoma, representing 91% of cases, with a minor proportion exhibiting predominant adenocarcinoma with neuroendocrine features (1.2%) (Figure 2). The patient’s haematological and biochemical baseline parameters, tumour grading and further characteristics are shown in Tables 1 and 2. The PSA in the entire cohort ranged from 0.07 to 3,400 ng/mL with a mean PSA of 174 (± 981.48 SD). The majority of the patients (51.3%) had Grade Group III–IV disease with a Gleason score of >8 was found in 67.5% of the population. Forty-one (51.25%) patients had undergone bilateral orchidectomy. The median time of GnRH analogue use was 32 months. Most of the patients (72.5%) were castration sensitive.

Low (C and D) and high power (A and B) images show prostate parenchyma involved by a neoplastic lesion exhibiting glandular crowding and infiltrative growth pattern. Individual neoplastic cells show nuclear enlargement and nucleolar prominence.

**Figure 3.**
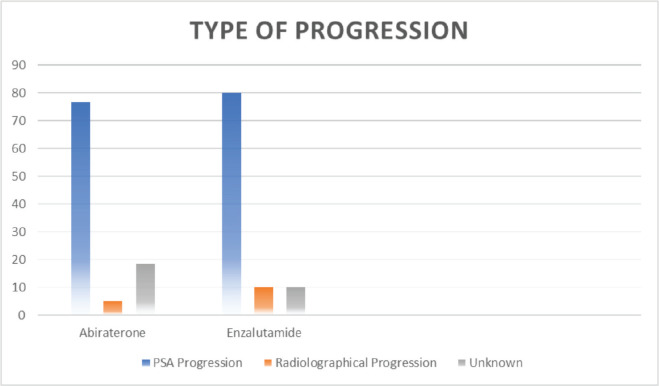
Type of progression.

Out of 80 patients who fulfilled the study criteria, 60 (75%) patients were treated with abiraterone and 20 (25%) patients were treated with enzalutamide (Figure 1). Progression types were primarily characterised by PSA progression (76.66% with abiraterone versus 80.0% with enzalutamide) and radiographical progression with stable PSA (5% with abiraterone versus 10% with enzalutamide) (*p* = 0.047) (Figure 3 and Table 3). Patients with PSA progression, were later followed by imaging studies and showed radiographical progression as well.

Dose modifications were implemented in 16% of patients receiving abiraterone compared to 7.75% with enzalutamide, although this difference was not statistically significant (*p* = 0.2). Our retrospective charts have limited documentation for dose modifications; however, available data revealed the most common cause of dose modifications was haematological toxicity (in both groups) and hepatotoxicity (with abiraterone). Bone metastasis emerged as the predominant site, accounting for 81.25% of the total cohort, while additional occurrences encompassed lung, liver and retroperitoneal nodal deposits. The utilisation of bone protective agents was widespread, with zolindronic acid administered in 71.25% of cases, followed by an equal number of patients receiving Denosumab and pamidronate. Most of the patients had a PSA doubling time of >6 months, 80% versus 75% in the abiraterone and enzalutamide groups, respectively (Table 3). A limited number of patients (26% in the abiraterone group versus 10% in the enzalutamide group) also received palliative local radiation.

**Figure 4.**
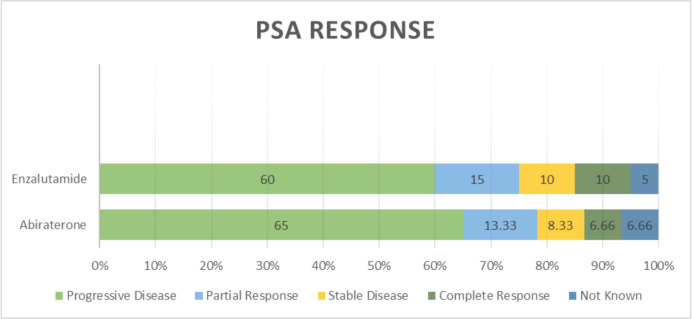
Types of PSA response.

PSA response rates varied, with both drugs (abiraterone and enzalutamide) showing similar proportions of PD (60% versus 65%), PR (13.33% versus 15%), SD (8.33% versus 10.0%) and CR (6.66% versus 10.0%) (*p* = 0.036) (Table 2 and Figure 4). Follow-up data of available patients showed that patients who progressed were switched to either alternate hormonal agents or systemic chemotherapy (docetaxel), with 4 (5%) being transferred to palliative services with best supportive care only. Approximately half of the patients in the entire cohort were alive at the time of the last follow-up.

**Figure 5.**
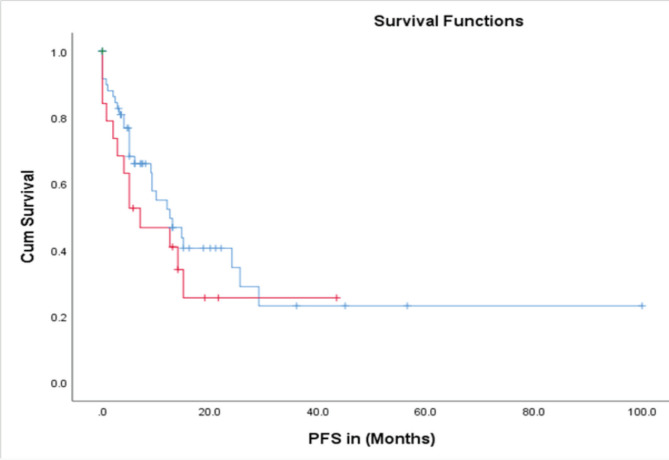
PFS in abiraterone (blue) versus enzalutamide (red) treatment arms.

**Figure 6.**
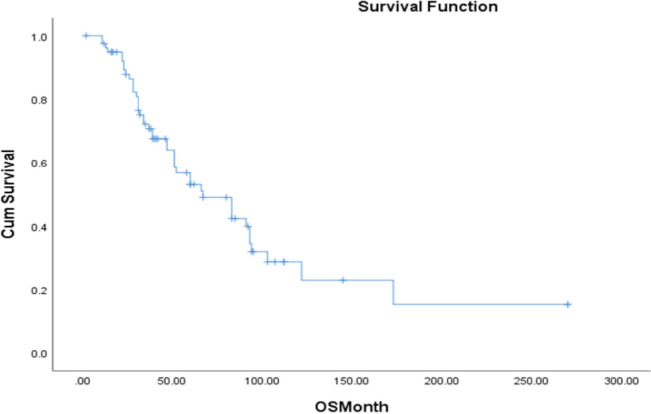
OS in the entire cohort.

The time to treatment progression (TTP) for the entire study was 19 months, with no difference in the median PFS between patients treated with abiraterone and enzalutamide i-e 19 versus 18 months (95% CI 9.7–27.9; *p* = 0.004) (Figure 5). The median OS in the entire cohort was 67 months (95% CI 39–94; *p* = 0.003) (Figure 6).

## Discussion

While a cure for metastatic prostate cancer remains out of reach, ongoing advances in management have significantly improved patient outcomes. Current therapeutic options, often used in combination, include hormonal therapies, chemotherapy, bisphosphonates, lutetium-177 PSMA therapy, targeted therapies with PARP inhibitors and immunotherapy. Despite these advancements, initial treatment with oral hormonal agents combined with ADT remains the preferred approach for treatment-naive metastatic prostate cancer, especially alongside systemic chemotherapy in cases with extensive bone involvement. Treatment decisions are influenced by factors such as life expectancy, comorbidities, patient preferences and tumour characteristics.

Abiraterone and enzalutamide are the cornerstone of the treatment of metastatic prostate carcinoma. There are currently no head-to-head comparative prospective clinical trials to prefer one over the other. Abiraterone acetate, an androgen biosynthesis inhibitor, has shown substantial efficacy in several key trials. The COU-AA-302 trial evaluated 1,088 patients who had not received prior chemotherapy and demonstrated a significant improvement in radiographic PFS with a median of 16.5 months for the abiraterone group compared to 8.3 months for the placebo group. Similar results have been demonstrated in our study where median PFS was found to be 19 months in the abiraterone group. Furthermore, the median OS in COU-AA-302 trial was significantly longer in the abiraterone acetate group than in the placebo group (34·7 months (95% CI 32·7–36·8) versus 30·3 months [28·7–33·3]; hazard ratio 0·81 (95% CI 0·70–0·93); *p* = 0·0033) [6]. COU-AA-301 trial focused on 1,195 patients’ post-docetaxel treatment and similarly demonstrated significant benefits. The median PFS was 5.6 months for the abiraterone group versus 3.6 months for the placebo group, and the median OS was 15.8 months for the abiraterone group compared to 11.2 months for the placebo group. These results confirm the role of Abiraterone in extending survival and delaying disease progression in patients with metastatic castration-resistant prostate cancer (mCRPC) [7].

In high-risk mCSPC, LATITUDE trial provided further evidence of abiraterone efficacy. This trial included 1,199 patients and showed that the median OS was significantly longer in the Abiraterone group (53.3 months) compared to the placebo group (36.5 months). In our study analysis, the median OS in the entire cohort was 67 months. Additionally, the LATTITUDE trial reported improvements in secondary endpoints such as pain progression, skeletal-related events and PSA progression [8]. Our study also demonstrated good tolerance of abiraterone with significant improvement in disease symptoms with only 16% of the patients undergoing dose modifications in the abiraterone group.

Enzalutamide, an androgen receptor inhibitor, has also demonstrated significant efficacy in various clinical settings. The PREVAIL trial, which included 1,717 chemotherapy-naive patients, demonstrated a median PFS of 20.0 months for the Enzalutamide group. Our study yielded similar findings, with a median PFS of 18 months in the enzalutamide treatment arm. Moreover, in the PREVAIL trial, the median OS was 36 months for the Enzalutamide group compared to the 31-month median OS for the placebo group, indicating a substantial survival benefit. Additionally, enzalutamide was associated with a high PSA response rate (78%) and significant delays in radiographic progression and death [9].

The AFFIRM trial targeted 1,199 patients, post-chemotherapy and revealed that the median PFS was 8.3 months for the Enzalutamide group versus 3.0 months for the placebo group. The median OS was 18.4 months for the Enzalutamide group compared to 13.6 months for the placebo group. These results show enzalutamide effectiveness in prolonging survival and managing disease progression in mCRPC [10]. The ENZAMET trial also evaluated Enzalutamide in combination with testosterone suppression in 1,125 patients with metastatic hormone-sensitive prostate cancer. The trial reported significantly longer PFS and OS for the Enzalutamide group compared to the standard nonsteroidal antiandrogen therapy group, although the incidence of seizures and other toxic effects was higher in the Enzalutamide group. This highlights the importance of balancing efficacy and safety in clinical decision-making [11]. Another trial, ARCHES included 1,150 patients with mHSPC and showed that the median rPFS was not reached for the Enzalutamide group compared to 19.0 months for the placebo group. The trial confirmed enzalutamide’s ability to significantly reduce the risk of metastatic progression or death, with a safety profile consistent with previous trials [12]. Therefore, both abiraterone and enzalutamide are preferred first-line treatments for metastatic prostate adenocarcinoma due to their significant survival benefits.

Our study showed a comparable TTP with abiraterone and enzalutamide treatment arms with no major clinically significant difference in the survival benefits between the two groups (median PFS of 19 versus 18 months). These findings are consistent with previous clinical trials that have established the efficacy of these agents in prolonging PFS in mCRPC patients. Despite the predominance of high Gleason grades (III/IV) and Gleason scores greater than 8 in our cohort, the median OS was notably extended to 67 months, potentially reflecting differences in patient populations, treatment protocols and healthcare settings.

PSA response rates in our study varied, with both abiraterone and enzalutamide showing similar proportions of PD, PR, SD and CR. There was no statistically significant difference between the two treatment groups suggesting comparable efficacy in achieving PSA control. This aligns with the results from other real-world studies that have shown similar PSA response rates for both agents. Progression types were primarily characterised by PSA progression and radiographic progression, with no significant difference observed between the two treatment groups; PSA progression of 77% with abiraterone versus 80% with enzalutamide (*p* = 0.047). This highlights the importance of regular monitoring of PSA levels and imaging studies to assess disease progression and guide treatment decisions.

The high prevalence of comorbid conditions, such as diabetes (36%), HTN (57%) and IHD (30%), shows the complexity of managing metastatic prostate cancer in real-world settings. The safety profiles of abiraterone and enzalutamide were comparable, with dose modifications implemented in 16% of patients receiving abiraterone and 7.75% with Enzalutamide, although this difference was not statistically significant (*p* = 0.2).

Apart from clinical trials, there are multiple retrospective analysis done on the utility of enzalutamide and abiraterone in metastatic prostate cancer. A recently published retrospective analysis also showed similar PFS (20.6 m for abiraterone versus 22.5 m for enzalutamide). The median OS was notably shorter for patients treated with abiraterone compared to those treated with enzalutamide. This difference was observed in patients aged 75 and older, white patients, those with pre-existing conditions such as diabetes, cardiovascular disease, both diabetes and cardiovascular disease, renal disease and across all socioeconomic levels [13]. Real-world data from abiraterone and enzalutamide show similar activity in different populations; however, there is no published data on its efficacy in the Pakistani population [14, 15]. Therefore, our study confirmed that abiraterone and enzalutamide exhibit comparable efficacy in the Pakistani population, similar to findings from studies on Western populations.

Our study has several limitations that should be acknowledged. First, the retrospective design may introduce selection bias and limit the generalisability of our findings. Second, the relatively small sample size and single-centre setting may affect the robustness of the results.

Given the retrospective nature and the specific small patient population size, the primary aim of our study was to evaluate real-world clinical outcomes of metastatic prostate adenocarcinoma patients treated with abiraterone or enzalutamide, rather than to establish definitive comparative efficacy. For future research, we agree that conducting prospective studies with larger sample sizes and predefined power calculations would be beneficial to validate our findings and provide more definite conclusions regarding the differences between the two arms. Furthermore, side effects and safety data were not documented properly in the patient charts reviewed. Despite its limitations, our study demonstrated that both hormonal agents are equally effective when used as preferred treatment agents in patients with metastatic prostate adenocarcinoma in our population.

## Conclusion

Our study demonstrated that both abiraterone and enzalutamide are effective in prolonging PFS and OS in metastatic prostate adenocarcinoma patients. These findings contribute to the growing body of evidence supporting the use of these hormonal therapies in real-world clinical practice. Further prospective studies with larger cohorts and multi-centre designs are warranted to validate our findings and refine treatment strategies for metastatic prostate cancer.

## Conflicts of interest

All authors declare no competing financial interests.

## Funding

No funding was available.

## Ethical approval

The study was approved by the ERC of the Aga Khan University Hospital (AKUH), Karachi, Pakistan (ERC No: 8789-24932).

## Consent to participate

Due to the retrospective nature of the study, the need for informed consent was waived by the ERC of Aga Khan University Hospital, Karachi, Pakistan.

## Consent to publication

Not applicable.

## Availability of data and materials

The data that has been used is confidential. The datasets are available from the corresponding author.

## Author contributions

MY and SR: Designed and wrote the manuscript.

HI, IA, AH, MR: Collected the data and performed the experiment.

NZ: Analysed the data.

YR, MM: Reviewed and edited the manuscript.

SR: Supervised the manuscript.

## Figures and Tables

**Table 1.** Baseline characteristics of the study population.

Variables	Total = 80
Age (median)	65 (100%)
ECOG • 0–2 • 2–4	71 (88%) 09 (12%)
Diabetes	29 (36%)
HTN	46 (57%)
IHD	24 (30%)
Others	12 (15%)
Family history • Breast • Lung • Prostate • Pancreas • Others	10 (12.5%) 2 (2.5%) 1 (1.25%) 2 (2.5%) 1 (1.25%) 4 (5%)
Genetic test performed	11 (13.25%)
Known BRCA mutation	2 (18.2%)
Mutation of unknown significance	4 (36.36%)
History of prior radical prostatectomy	6 (7.5%)
Haemoglobin (g/dL)	12.09 (± 3.04 SD)
WBC (×10^9^/L)	10.5 (±5.8 SD)
Platelets (×10^9^/L)	252 (±94 SD)
NLR	5.03 (±3.19 SD)
PLR	21.5 (±80.9 SD)
Calcium (mg/dL)	10.2 (±20.3 SD)
Vitamin D (ng/mL)	8.1 (±9.9 SD)
PSA (ng/mL)	174 (± 981.48 SD)
Total Bilirubin (mg/dL)	0.8 (±3.1 SD)
ALT (IU/L)	41 (±65 SD)
LDH (U/L)	190 (±130 SD)
Serum testosterone (ng/dL)	28.69 (± 96.73 SD)
Albumin (g/dL)	3.85 (± 0.70 SD)

SD = Standard deviation

**Table 2.** Disease characteristics of the study population.

Tumour characteristics	Total = 80 (100%)	*p*-value
Grade group		0.95
Grade I	29 (36%)	
Grade II	2 (2.5%)	
Grade III	9 (11.3%)	
Grade IV	32 (40%)	
Not known	08 (10%)	
Histopathology Adenocarcinoma Predominant adenocarcinoma with neuroendocrine features Not documented/Missing data	72 (90%) 01 (1.2%) 07 (8.7%)	0.91
Gleason score		0.90
7	07 (8.8%)	
8	13 (16.2%)	
>8	54 (67.5%)	
Not known	06 (7.5%)	
Gleason pattern 3 + 4 4 + 3 4 + 4 4 + 5 5 + 3 5 + 4 5+5 Not known/Not performed	04 (5%) 11 (13.8%) 25 (31.3%) 11 (13.8%) 3 (3.8%) 11 (13.8%) 7 (8.8%) 8 (10%)	0.99

**Table 3.** Abiraterone and enzalutamide treatment arms.

Variable	Abiraterone *N* = 60 (%)	Enzalutamide *N* = 20 (%)	*p*-value
Progression type • PSA progression • Radiographical Progression • Not known/Not documented	46 (76.66%) 03 (5.0%) 11 (18.33%)	16 (80%) 02 (10%) 02 (10%)	0.047*
PSA doubling time • <6 months • >6 months • Not known/Not documented	08 (13.33%) 48 (80.00%) 04 (6.66%)	04 (20.00%) 15 (75.00%) 01 (5.00 %)	0.09
PSA response • PD • PR • SD • CR • Not known/Not documented	39 (65.00%) 08 (13.33%) 05 (8.33%) 04 (6.66%) 04 (6.66%)	12 (60.00%) 03 (15.0%) 02 (10.0%) 02 (10.0%) 01 (5.0%)	0.036*
Local radiation received	21 (26%)	08 (10%)	-
Site of metastasis • Bone only • Lung + Bone • Liver + Bone • Nodal + Bone • Retroperitoneal nodal only • Others	39 (65.0%) 02 (3.33%) 01 (1.66%) 06 (10.0%) 09 (15.0%) 03 (5.0%)	13 (65.0%) 02 (10.0%) 02 (10.0%) 00 03 (15.0%) 00	0.76
Bone protective agents used • Zoledronic • Denosumab • Pamidronate • None	42 (70.0%) 05 (8.33%) 06 (10.0%) 07 (11.66%)	15 (75.0%) 01 (5.00%) 02 (10.00%) 02 (10.00%)	0.97
Orchidectomy	32 (53.33%)	09 (45.0%)	-
Follow-up Dead Alive Lost to follow-up	23 (38.33%) 30 (50.00%) 07 (11.66)	09 (45.00%) 11 (55.00%) 00	0.32

* Statistically significant
